# Supplementary material for: Electrical control of spins and giant g-factors in ring-like coupled quantum dots
Source: Nat Commun. 2019 Dec 16;10:5740. doi: 10.1038/s41467-019-13583-7 (PMC6915759; doi:10.1038/s41467-019-13583-7)
Supplement: Supplementary file 1 — Supplementary Information [file 41467_2019_13583_MOESM1_ESM.pdf]

Supplementary Information

Electrical control of spins and giant g-factors in ring-like coupled quantum dots

H. Potts[1†*], I–J. Chen[1†], A. Tsintzis[1], M. Nilsson[1], S. Lehmann[1], K. A. Dick[1,2], M. Leijnse[1], C. Thelander[1*]

[1] Division of Solid State Physics and NanoLund, Lund University, SE-221 00 Lund, Sweden

[2] Centre for Analysis and Synthesis, Lund University, SE-221 00 Lund, Sweden

[†] These authors contributed equally to the work. [*] heidi.potts@ftf.lth.se, claes.thelander@ftf-lth.se

Supplementary Notes

Supplementary Note 1: General information about sample A

Supplementary Figures 1a-b show overview measurements of sample A at V_{BG} = 1 V, and V_{BG} = -1 V, respectively. The orbital crossings which are presented in this supporting material are highlighted. As in the main article, we label the crossings (O_L,O_R), where O_L and O_R is the orbital number of the left and right QD. Crossings (2,3), (1,1) and (2,2) were investigated at V_{BG} = 1 V, while crossing (4,1) and crossing (5,2) were studied at V_{BG} = 0 V, and -1.5 V, respectively. Since changing V_{BG} affects the tunnel couplings between the left and right QD, we note that some crossings are only clearly visible for specific gate voltage ranges.

The behaviour of two isolated orbitals is presented in Supplementary Figures 1c-f. We focus on the 2nd orbital from the left QD, and the 3rd orbital from the right QD, which are the relevant orbitals for crossing (2,3). Supplementary Figure 1c shows transport as a function of V_L for O_L = 2, when there are zero electrons in the right QD. The charging energy E_c and lever arm $\alpha_{VL/L}$ of V_L on the left QD can be extracted from the height and the width of the Coulomb diamond. The lever arm $\alpha_{VR/L}$ of V_R on the left QD can then be calculated using the slope of the conductance lines Supplementary Figure 1a. Similar analysis can be done for the right QD using Supplementary Figure 1e, and all parameters for both QDs are shown in Supplementary Table 1.

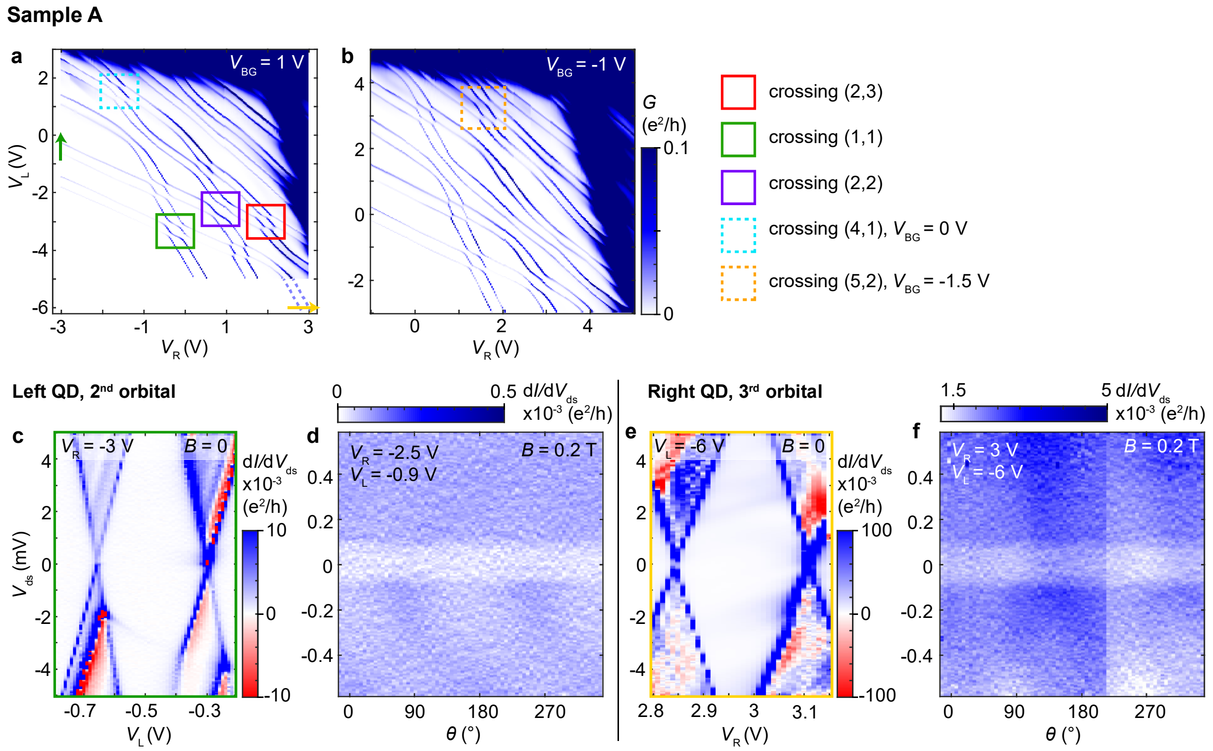

Supplementary Figure 1 **Overview measurements and behaviour of single orbitals. a-b** Conductance measurements over large sidegate voltage ranges for V_{BG} = 1 V, and -1 V, respectively. The crossings which are discussed in this supporting material are highlighted. **c** Measurement of dI/dV_{ds} versus V_{ds} as a function of V_L for the 2nd orbital of the left QD (green vector). **d** Measurement of dI/dV_{ds} versus V_{ds} as a function of B-field direction. **e-f** Corresponding measurements for the 3rd orbital of the right QD.

	E_c (meV)	$\alpha_{VL/L}$ (meV/V)	$\alpha_{VR/L}$ (meV/V)	$\alpha_{VL/R}$ (meV/V)	$\alpha_{VR/R}$ (meV/V)
Left QD	9.9	28	19	-	-
Right QD	7.6	-	-	11	29

Supplementary Table 1 Charging energies and leverarms of the QDs of sample A.

Knowing the lever arms, an orbital separation > 30 meV for both QDs can be calculated from Supplementary Figure 1a. Finally, we note that the Zeeman splitting of the single QD orbitals show almost no rotation dependence (Supplementary Figures 1d,f), and a g^* of ~ 8 can be calculated for both orbitals.

Supplementary Note 2: Additional information about crossing (2,3) of sample A

The main article shows the most important transport characteristics of crossing (2,3). Here we present additional data from the same crossing.

1e regime

We start by showing data from the 1e regime. To facilitate the discussion, we present the numerical calculation of the state energies as function of $B_{||}$ again in Supplementary Figure 2a (same as Figure 2b in the main article). Supplementary Figures 2b-c show transport as a function of B-field orientation for B = 0.05 T, and B = 0.5 T (B = 0.2 T is presented in the main article). While a large anisotropy of g^* can be observed for any $B_{||} > 0$, it is interesting to note the change in bowing of the smallest energy gap when going to higher B-fields. This is a consequence of a change in orbital momentum sign of the first excited state when the magnetic flux that penetrates the ring is sufficiently large. Supplementary Figures 2d-h show transport as a function of detuning in the 1e regime for different B-field strength. This series visualizes the hybridization of the 1st and the 2nd excited states (ES1, ES2) at zero detuning. For $B_{||} < 55$ mT, the gap between ES1 and the ground state (GS) increases with the B-field, and allows to extract g_1^* directly (which was done in the main article). For $B_{||} > 55$ mT the gap at zero detuning decreases with increasing B-field, since the two lowest states now have the same orbital momentum but the spin of excited state is favorable at higher fields. Eventually, for $B_{||} > 0.35$ T, the ground state spin of the ring changes and is the same as for the single QD orbitals. Therefore, no change in ground state is observed when detuning the orbitals at $B_{||} = 0.5$ T (Supplementary Figure 2h).

Using the lever arms we can now also convert $\Delta V_{L,R}$ to an energy, which can then be compared with Δ_{orb} from the simulations. In Supplementary Figures 2d-f, the left sidegate is changed from -3.15 V to -3.32 V, and simultaneously the right sidegate is changed from 1.74 V to 1.9 V.

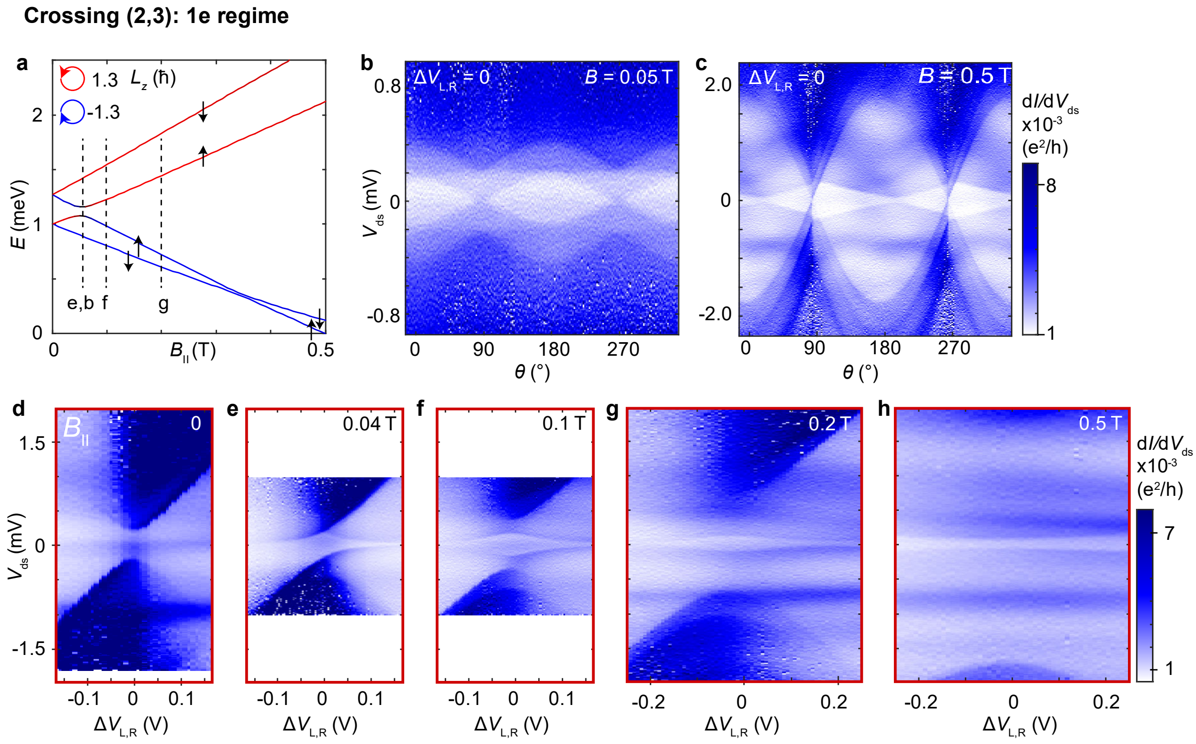

Supplementary Figure 2 **Additional transport data from the 1e regime of crossing (2,3). a** Numerical calculation of state energies as a function of $B_{||}$ (same as Figure 2b in the main article). **b-c** Measurement of dI/dV_{ds} versus V_{ds} as a function of B-field direction at zero detuning, for B = 0.05T, and 0.5T. **d-h** Measurement of dI/dV_{ds} versus V_{ds} recorded along the red detuning vector in the 1e regime, for different $B_{||}$-field strengths.

3e regime

As explained in the main article, the 3e regime shows a different behavior compared to the 1e regime. In Supplementary Figure 3 we present additional data supporting this observation. Transport as a function of $B_{\perp}$ (Supplementary Figure 3a) shows a very weak B-field dependence, similar to what has been shown for the 1e regime. A corresponding suppression of g^* at zero detuning can be observed in Supplementary Figure 3b. However, the magnetic field rotation for B = 0.2 T (Supplementary Figure 3b) shows a clearly different behavior, in particular there is no opposite bowing for the ES1-GS gap. This can also be observed in the B-field series of the detuning measurements (Supplementary Figures 3d-g): the gap at zero detuning continuously increases with $B_{||}$-field strength, and is always larger compared to the gap of the single orbitals. This also means that a change of the spin ground state (Figure 4d of the main article) does not occur in the 3e regime, neither as function of $B_{||}$ nor detuning.

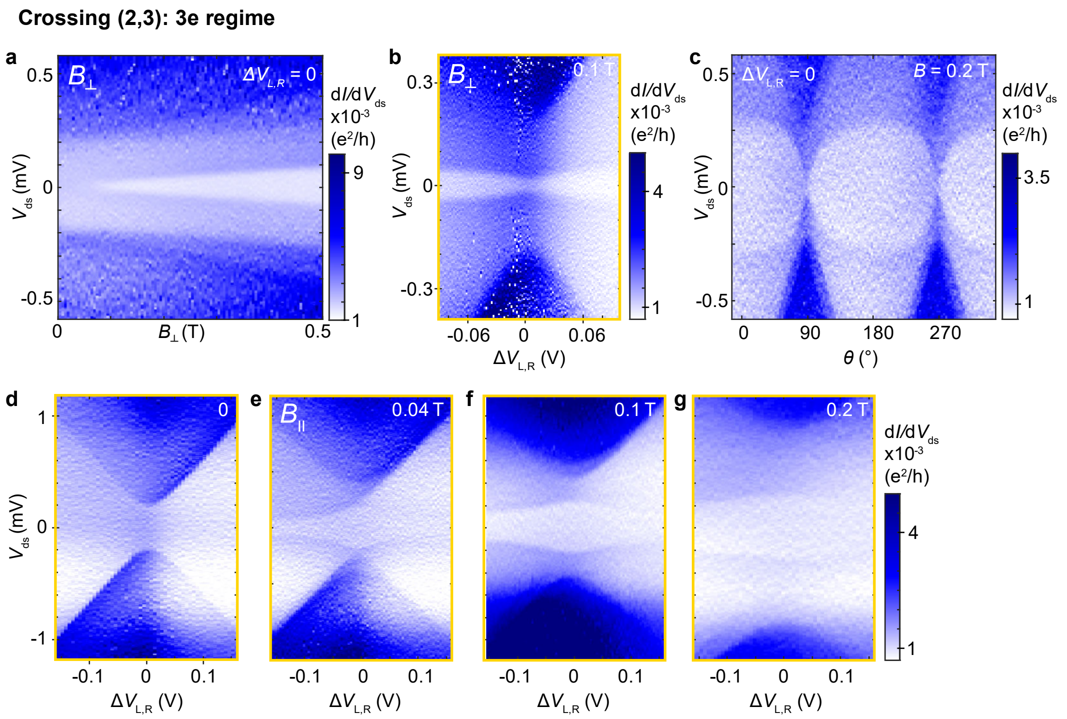

Supplementary Figure 3 **Additional transport data from the 3e regime of crossing (2,3). a-c** Measurement of dI/dV_{ds} versus V_{ds} as a function of $B_\perp$ at zero detuning. **d-i** Measurement of dI/dV_{ds} versus V_{ds} recorded along the yellow detuning vector in the 3e regime, for $B_\perp = 0.1$ T. **c** Measurement of dI/dV_{ds} versus V_{ds} as a function of B-field direction at zero detuning for B = 0.2 T. **d-g** Measurement of dI/dV_{ds} versus V_{ds} recorded along the yellow detuning vector in the 1e regime, for different $B_{||}$ -field strengths.

B-field dependence of the honeycomb diagram

Finally, we present how the ground states of crossing (2,3) depend on the B-field strength and orientation (Supplementary Figure 4). As highlighted in the main article, one characteristic of a crossing where the hybridization leads to the formation of ring-like states, is that the corners of the honeycomb appear very sharp. The strong suppression of the hybridization gap is suggested to be a consequence of tunnel-coupling an even and an odd QD orbital in two points. Another characteristic is that the energy of the ground states dramatically changes when increasing $B_{||}$ from 0 to 0.5 T (Supplementary Figures 4a-d). This stands in sharp contrast to Supplementary Figure 4e, which shows that $B_\perp = 0.5$ T effectively leaves the states unchanged compared to B = 0. We would like to highlight that this observation provides an easy way to screen for the occurrence of ring-like states in a honeycomb diagram: ring-like states can be identified by comparing the honeycomb pattern of the crossing at both parallel and perpendicular magnetic field.

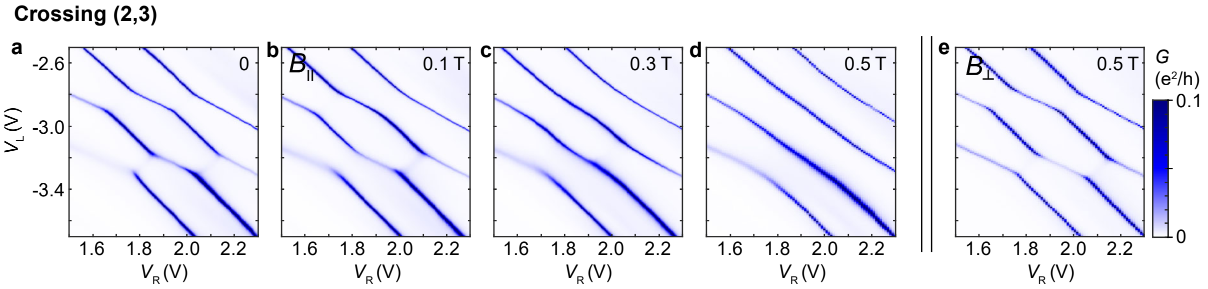

Supplementary Figure 4 **B-field dependence of crossing (2,3). a-d** Conductance as a function of sidegate voltages for $B_{\|} = 0$, 0.1 T, 0.3 T, and 0.5T, respectively. **e** Corresponding measurement for $B_{\perp} = 0.5$ T.

Supplementary Note 3: Ring states in other even/odd or odd/even crossings sample A)

Supplementary Figure 5 shows transport data from another even/odd and an odd/even crossing. In the overview measurements (Supplementary Figures 5a,f) very sharp corners can be observed, similar to crossing (2,3), which is related to the strongly suppressed hybridization gap. We study the 3e regime of crossing (4,1), and the 1e regime of crossing (5,2) in more detail. For both crossings, transport at zero detuning as a function of $B_{\|}$ (Supplementary Figures 5b,g) shows two excited states that rapidly increase in energy, and a huge anisotropy upon magnetic field rotation can be observed (Supplementary Figures 5c,h), corresponding to a large orbital contribution to the effective *g*-factor (g^{*}~ 50-75 for crossing (4,1), and g^{*}~ 35-43 for crossing (5,2)). Supplementary Figures 5d-e and 5i-j show transport as a function of detuning for different B-field. ES2 and ES3 quickly increase in energy with increasing B-field at zero detuning, as expected for ring states. For crossing (4,1) the detuning dependence of the GS-ES1 gap is very small (Supplementary Figure 5e), which implies that the hybridization of ES1 and ES2 occurs at very small $B_{\|}$-fields due to a small Δ_{SOI}. The GS-ES1 gap for the 1e regime of crossing (5,2) (Supplementary Figure 5j) shows a similar behavior compared to the 3e regime of crossing (2,3): the gap is the largest at zero detuning, and no ground state change occurs when increasing the detuning. This implies an opposite spin-filling sequence compared to crossing (2,3). However, the small splitting of the Kramers pairs makes it difficult to resolve all the states in the $B_{\|}$ sweep, and we therefore discuss this effect in more detail for Sample B.

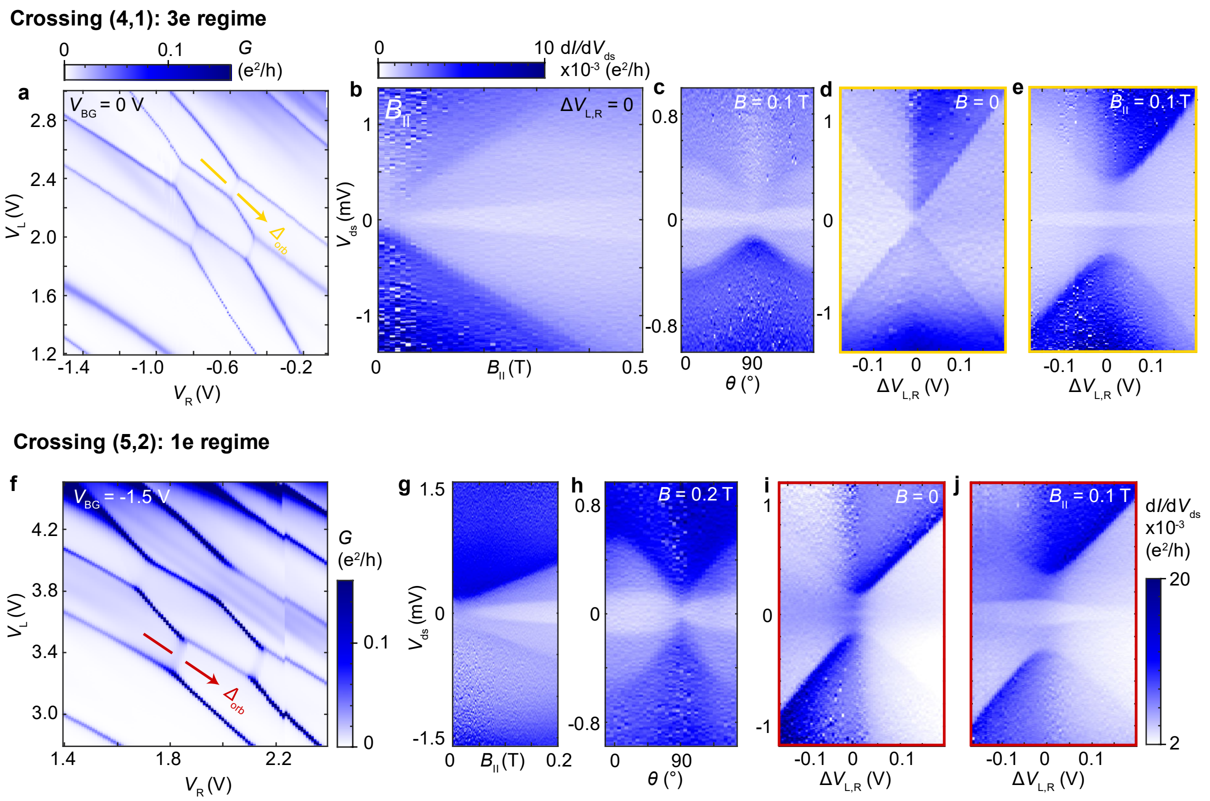

Supplementary Figure 5 **Transport in crossings (4,1) and (5,2). a** Conductance of crossing (4,1) as a function of sidegate voltages. **b-c** Measurement of dI/dV_{ds} versus V_{ds} at zero detuning in the 3e regime as a function of $B_{\parallel}$ and as a function of B-field direction. **d-e** Measurement of dI/dV_{ds} versus V_{ds} recorded along the red detuning vector in the 1e regime, for $B = 0$, and $B_{\parallel} = 0.1$ T, respectively. **f-j** Corresponding measurements for crossing (5,2) in the 1e regime.

Supplementary Note 4: Absence of ring states in even/even and odd/odd crossings (sample A)

Our theoretical models predict that perfect rings can only form in the case where and even and an odd orbital of the two QDs are involved. In the case of even/even and odd/odd crossings, the overlap integrals at the barriers have the same sign, leading to a significant hybridization gap. In Supplementary Figure 6 we present transport data from crossings (1,1) and (2,2) to support this statement. For both crossings, the overview conductance measurements (Supplementary Figures 6a,d) look comparable to measurements of strongly tunnel-coupled parallel QDs, which stands in contrast to what has been observed for the crossings discussed in this work so far. In Supplementary Figures 6b,e we present transport in the 1e regime as a function of B-field direction at zero detuning, and observe that there is almost no rotation dependence of the GS-ES transitions, which is in agreement with no orbital contribution to g^*. Accordingly, no change of the gap between GS and ES1 is found when detuning the orbitals along the red gate vector for $B_{\parallel} = 0.2$ T (Supplementary Figures 6c,f).

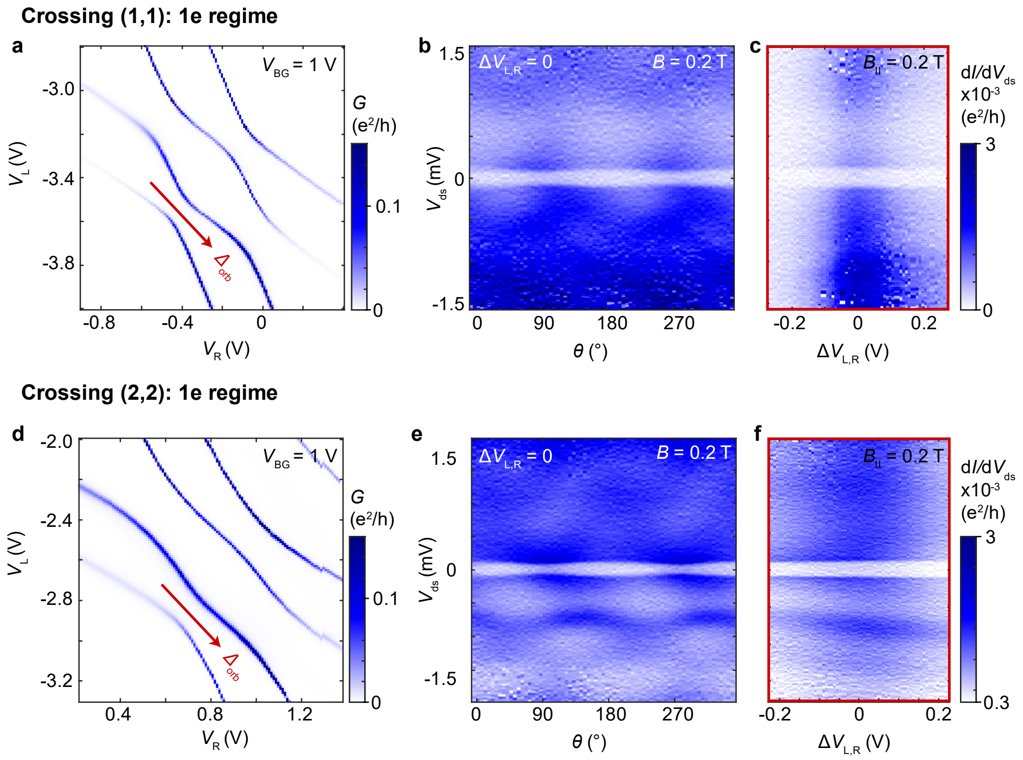

Supplementary Figure 6 **Transport in crossings (1,1) and (2,2). a** Conductance of crossing (1,1) as a function of sidegate voltages. **b** Measurement of dI/dV_{ds} versus V_{ds} at zero detuning in the 1e regime as a function of B-field direction. **c** Measurement of dI/dV_{ds} versus V_{ds} recorded along the red detuning vector in the 1e regime for $B_{\parallel}$ = 0.2 T. **d-f** Corresponding measurements for crossing (2,2).

Supplementary Note 5: Ring states in sample B

The emergence of ring states could be reproduced in a second sample (Sample B), with design similar to Sample A. An overview conductance measurement of Sample B is presented in Supplementary Figure 7a, and the relevant crossing is shown in Supplementary Figure 7b. Transport along the green gate vector at $B_{\parallel}$ = 0.1 T (Supplementary Figure 7c) shows weakly outlined Coulomb diamonds, and inelastic co-tunneling transport in the 1e, 2e, and 3e regimes. The gap energy in the 1e regime corresponds to a $g^* \approx$ 43, which is much larger compared to the effective g-factor of bulk InAs, similar to what has been shown for sample A. In Supplementary Figures 7d-g we study the 1e regime in more detail. Transport at zero detuning as a function of $B_{\parallel}$ (Supplementary Figure 7d) shows a strong increase of the ES1-GS energy gap with increasing B-field, and an anti-crossing with ES2 can be observed at $B_{\parallel} \approx$ 0.15T. A huge anisotropy upon magnetic field rotation can be observed in Supplementary Figure 7e, corresponding to a large orbital contribution to g^*.

Detuning the orbitals along the red gate vector (Supplementary Figures 7f-g) shows a strong increase in the gap between GS and ES1 upon formation of the ring states at zero detuning. The gap energy at zero

detuning increases continuously with increasing $B_{\|}$-field, and no ground state spin change can be observed when detuning the orbitals. This behavior is similar to what has been observed for the 3e regime of sample A. To explain this finding, we would like to recall that the spin filling sequence of the ring-like states depends on the sign of the spin-orbit interaction. Crossing (2,3) of Sample A is an example of a crossing for which the ground state at $B = 0$ and zero detuning is spin-up, which is the unfavored spin-direction for high magnetic fields. This leads to a ground state spin-change with increasing $B_{\|}$ in the 1e regime, as well as a ground state spin change when detuning the orbitals at constant magnetic field. The 3e regime, on the other hand, does not show these features. The experimental results of Sample B can therefore be explained by an opposite spin-filling sequence, which is related to an opposite sign of the spin-orbit interaction.

In conclusion, Sample B shows the possibility to create ring-like states, very similar compared to Sample A. In addition, Sample B clearly shows an opposite spin-filling sequence compared to crossing (2,3) of Sample A. However, we would like to note that the spin-filling sequence seems not sample specific but rather depends on the properties of each individual crossing, as a similar behavior was already observed for crossing (5,2) of Sample A.

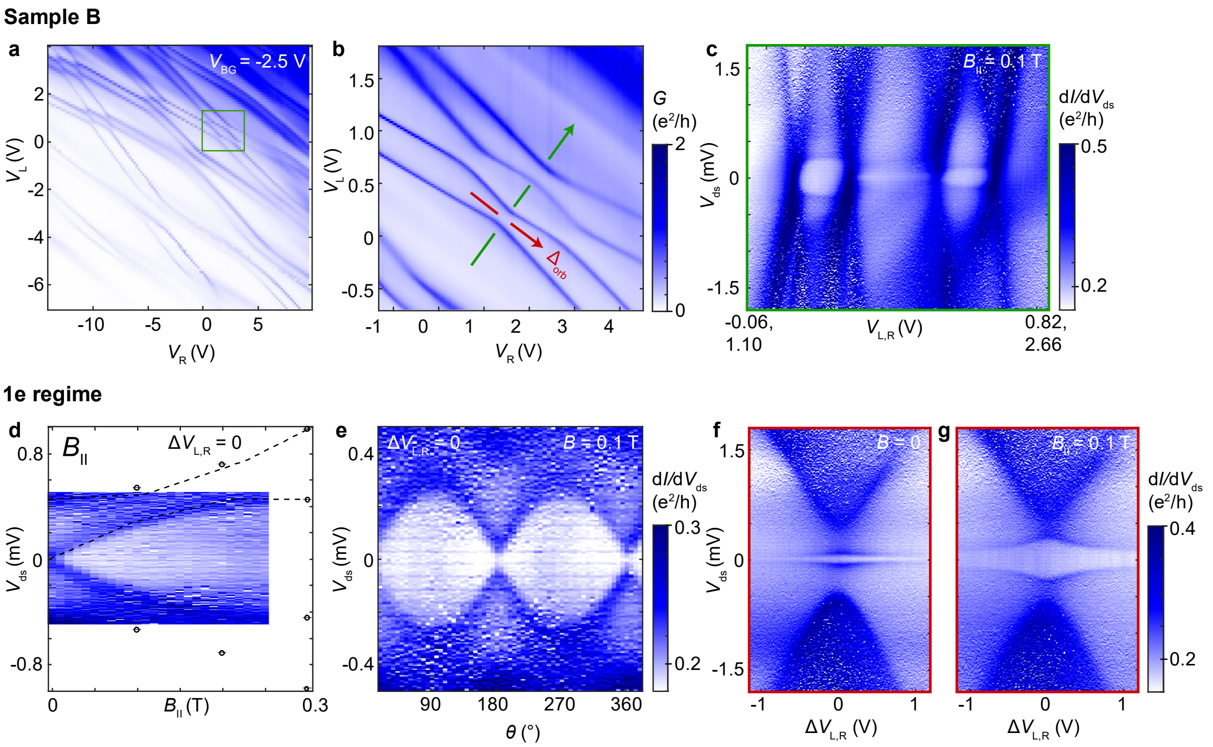

Supplementary Figure 7 **Transport of sample B. a** Conductance of Sample B as a function of sidegate voltages. **b** Overview measurement of the crossing which is investigated in more detail. **c** Measurement of dI/dV_{ds} versus V_{ds} along the green gate vector. **d-e** Measurement of dI/dV_{ds} versus V_{ds} at zero detuning in the 1e regime as function of $B_{\|}$ and as a function of B-field direction. The datapoints added to **d** represent gap energies extracted from detuning measurements at different $B_{\|}$-field. **f-g** Measurement of dI/dV_{ds} versus V_{ds} recorded along the red detuning vector in the 1e regime, for $B = 0$, and $B_{\|} = 0.1$ T, respectively. The finite gap around zero bias in the case of $B = 0$ can be explained by the ferromagnetic contacts of the device.

Supplementary Methods

Supplementary Methods 1: Three-dimensional numerical simulation of single-electron states

In this section, we discuss the theoretical model used in the numerical simulation of single-electron states under different electrostatic detuning and magnetic field. Under the effective mass approximation, the Hamiltonian of a single electron in a magnetic field can be expressed as[1]

$$H = \frac{(-\mathrm{i}\hbar\boldsymbol{\nabla}+e\mathbf{A})^2}{2m^*} + V(\mathbf{r}) + \frac{g_{\mathrm{spin}}^*}{2}\mu_{\mathrm{B}}\boldsymbol{\sigma}\cdot\mathbf{B} + H_{\mathrm{SO}}, \tag{1}$$

with electron effective mass m^*, elementary charge e, electron spin g-factor g_{spin}^*, Bohr magneton μ_{B}, Pauli vector $\boldsymbol{\sigma}$, magnetic vector potential $\mathbf{A} = (A_{\mathrm{x}}, A_{\mathrm{y}}, A_{\mathrm{z}})$, and magnetic field $\mathbf{B} = \boldsymbol{\nabla}\times\mathbf{A}$. $V(\mathbf{r})$ and H_{SO} are the electrostatic potential and the spin-orbit (SO) interaction term, respectively. $V(\mathbf{r})$ can be obtained by solving the Poisson equation

$$\boldsymbol{\nabla}\cdot\left(\varepsilon_{\mathbf{r}}\varepsilon_0\boldsymbol{\nabla}V(\mathbf{r})\right) = 0, \tag{2}$$

and the boundary condition $\mathbf{n}\cdot\boldsymbol{\nabla}(V_{\mathrm{out}} - V_{\mathrm{in}}) = \frac{\rho_{\mathrm{s}}}{\varepsilon_{\mathbf{r}}\varepsilon_0}$, with potential discontinuity at the surface $V_{\mathrm{out}} - V_{\mathrm{in}}$, an outward pointing normal vector $\mathbf{n}$, dielectric constant $\varepsilon_{\mathbf{r}}$, vacuum permittivity ε_0, and surface charge density ρ_{s}.

Here we consider Rashba-type spin-orbit coupling: an electric field in the direction $\mathbf{e}$ results in SO coupling of the form[1,2]

$$H_{\mathrm{SO,R}} = \frac{\alpha}{\hbar}(\mathbf{e}\times\mathbf{p})\cdot\boldsymbol{\sigma}, \tag{3}$$

where α is the Rashba parameter and $\mathbf{p}$ is the electron kinetical momentum. For the simulation results presented in the article, we assume the electric field to be along the nanowire axis $\mathbf{E} = E_0(0, 0, 1)$. Therefore, we can explicitly express the SO coupling term as

$$H_{\mathrm{SO}} = \alpha\left(\sigma_x\left(-\mathrm{i}\frac{\partial}{\partial y} + \frac{eA_y}{\hbar}\right) - \sigma_y\left(-\mathrm{i}\frac{\partial}{\partial x} + \frac{eA_x}{\hbar}\right)\right). \tag{4}$$

It is however worth noting that a quantitatively similar result was achieved with electric fields pointing in the nanowire radial direction as shown in Supplementary Table 2. In this case, we assume the electric field to be $\mathbf{E} = E_0\left(\frac{x}{R}, \frac{y}{R}, 0\right)$ and write the Rashba parameter as $\alpha^* = \left(\beta\frac{x}{R}, \beta\frac{y}{R}, 0\right)$, which depends on the spatial coordinates. R (= 40 nm) is the radius of the nanowire, and E_0 and β correspond to the amplitudes of the electric field and the Rashba parameter at the periphery of the quantum dot. As the

electron wave functions are not located exactly at the periphery of the quantum dot ($\sqrt{x^2+y^2} \leq R$), the effective Rashba parameter is therefore smaller than β. The SO interaction is expressed as

$$H_{\mathrm{SO,R}} = \beta\frac{x}{R}\left(\sigma_y\left(-\mathrm{i}\frac{\partial}{\partial z}+\frac{eA_z}{\hbar}\right)-\sigma_z\left(-\mathrm{i}\frac{\partial}{\partial y}+\frac{eA_y}{\hbar}\right)\right)+\beta\frac{y}{R}\left(\sigma_z\left(-\mathrm{i}\frac{\partial}{\partial x}+\frac{eA_x}{\hbar}\right)-\sigma_x\left(-\mathrm{i}\frac{\partial}{\partial z}+\frac{eA_z}{\hbar}\right)\right). \quad (5)$$

We solve the differential equations (Supplementary Equation 1 and 2) based on the finite-element method in COMSOL. First, we obtain $V(\mathbf{r})$ by solving Eq. S2 for the device structure shown in Supplementary Figure 8. Afterwards, $V(\mathbf{r})$ is used as an input and Supplementary Equation 1 is solved for the InAs nanowire structure colored in blue and green in Supplementary Figure 8. Here, the Poisson equation and the single electron Hamiltonian are not solved self-consistently, therefore electron-electron interaction is neglected and the calculated spectrum corresponds to the single-electron energy states of an empty quantum dot.

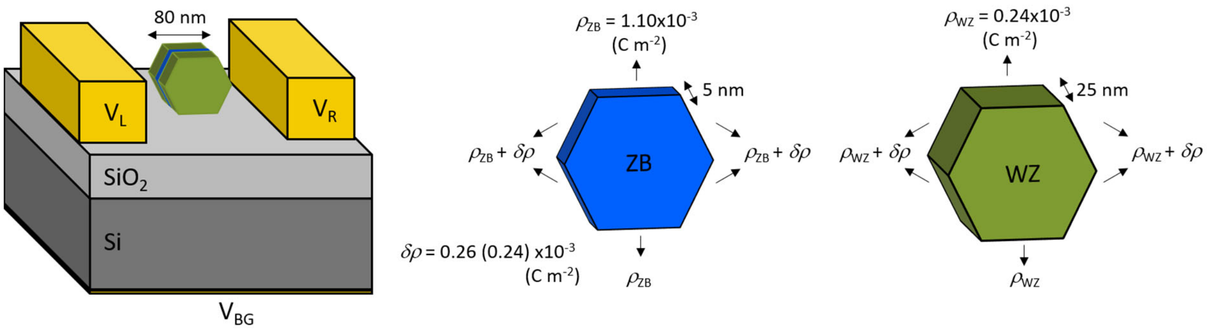

Supplementary Figure 8 **Device structure used in the numerical simulation**. The surface charge on the side and top/bottom facets in zinc blende ZB (blue) and wurtzite WZ (green) InAs are indicated. A lower surface charge is set for wurtzite as found in Ref. 3. An extra positive surface charge density of δρ = 0.26x10^{-3} C m^{-2} or 0.24x10^{-3} C m^{-2} is added to all the side facets in the simulation to fit the experimental data.

In the simulation an extra positive surface charge density δρ is assigned to the side facets, which leads to electrostatic potential barriers near the top and bottom facets and parallel-coupled double quantum dot states. Several parameters are tuned to match the state energies of the (2,3) crossing (at zero detuning, $\Delta_{\mathrm{orb}} = 0$) and their evolution with external magnetic field along the nanowire axis direction $B_{\|}$. More specifically, δρ and the gate configurations (set to approximately the experimental values) determine the circular symmetry of $V(\mathbf{r})$ in the nanowire and thus the backscattering of the orbiting electrons, which is characterized by the energy split δ (indicated by pink arrows in Supplementary Figure 9b). In addition, they also determine the orbital angular momentum L_z of the states. The angular motion of the electron couples to the external magnetic field and contributes to the g-factor with

$$g^*_{\mathrm{orbital}} = \frac{\mu_B}{m^*\hbar}L_z, \quad (6)$$

which increases with decreasing electron effective mass m^*. In the simulation, by adjusting the surface charges (ρ_{ZB} and ρ_{WZ}) and the zinc blende-wurtzite conduction band offset, we can modify the relative probability density distribution of the electronic states in the zinc blende ($m^* = 0.026\ m_e$[5]) and wurtzite ($m^* = 0.037\ m_e$[6]) segments to match the simulated g^*_{orbital} (at given δ and L_z) with the experimental values. The effective g-factor (g^*) is approximately given by $g^* \approx g^*_{\mathrm{spin}} + g^*_{\mathrm{orbit}}$, and therefore $g^*_{\mathrm{spin}} \approx (g_1^* + g_2^*)/2$. Finally, $\alpha\,(\alpha^*)$ is adjusted to match the energy gap at $B_{\|} = 0$ ($\Delta E_{B=0}$, indicated by blue arrows in Supplementary Figure 9b) and the value of $B_{\|}$ when states with opposite signs of L_z anti-cross (indicated by pink arrows). The calculated values of δ, $\Delta E_{B=0}$, g_1^*, g_2^*, and the relevant material parameters are shown in Supplementary Tables 2 and 3. The same parameters are used to simulate the evolution of the state energies at different detuning and external magnetic field as shown in the main text. In the main text we show the calculated state energies with Rashba SO coupling induced by an electric field along the nanowire axis.

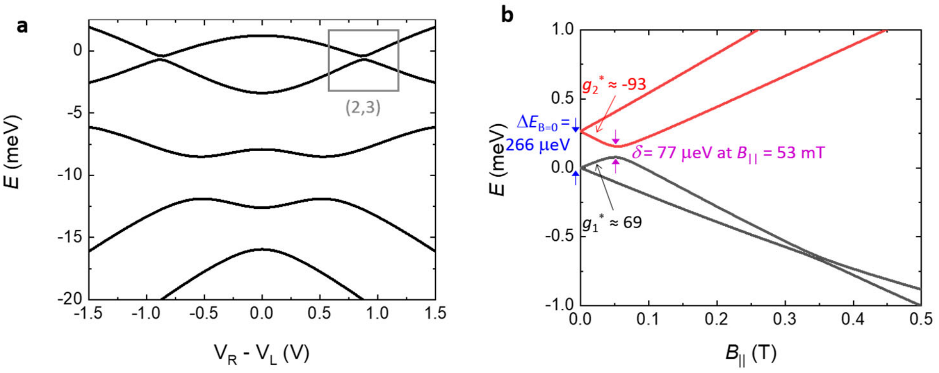

Supplementary Figure 9 **Numerical simulation of single-electron states. a** The lowest energy states as functions of the difference of the side gate voltages $V_R - V_L$. Here, $\mathbf{B} = 0$ and the spin states are therefore degenerate. The states at the (2,3) crossing (marked by the gray rectangle) are studied in detail for comparison with the experiment. **b** The states evolve with external magnetic field along the nanowire axis $B_{\|}$. Fitting parameters are tuned to match the energy gap at $B_{\|} = 0$ ($\Delta E_{B=0}$, indicated by blue arrows), the energy split related to backscattering δ and the anti-crossing $B_{\|}$ field (indicated by purple arrows), and the g-factors g_1^* and g_2^* between the experiment and the simulation.

	Experiment (Fig. 2a)	Simulation $(\mathbf{E} = E_0(0,0,1))$	Simulation $\left(\mathbf{E} = E_0\left(\frac{x}{R},\frac{y}{R},0\right)\right)$
δ (μeV)	50 at $B_{\|} \approx 55$ mT	77 at $B_{\|} = 53$ mT	75 at $B_{\|} = 50$ mT
$\Delta E_{B=0}$ (μeV)	240	266	260
g_1^*	59	69	74
g_2^*	-83	-93	-99

Supplementary Table 2 Comparison between the energy splits and effective g-factors extracted from the experiment and the numerical simulations based on assumptions that the SO coupling is induced by $\boldsymbol{E} = E_0(0,0,1)$ and $\boldsymbol{E} = E_0\left(\frac{x}{R},\frac{y}{R},0\right)$, respectively.

	Simulation ($\mathbf{E}=E_0(0,0,1)$)	Simulation $\left(\mathbf{E}=E_0\left(\frac{x}{R},\frac{y}{R},0\right)\right)$
$\delta\rho$ (C m^{-2})	0.26 ×10^{-3}	0.24 ×10^{-3}
ρ_{ZB} (C m^{-2})	1.10 ×10^{-3}	1.10 ×10^{-3}
ρ_{WZ} (C m^{-2})	0.24 ×10^{-3}	0.24 ×10^{-3}
ZB/WZ conduction band offset (meV)	120	120
g^*_{spin}	-11	-11
α (meV nm)	16.3	
β (meV nm)		4.2

Table 3 Material parameters which are used in the numerical simulation.

Supplementary Methods 2: Tight-binding model of a quantum ring

In this section, we describe an alternative way to model the two half-ring QDs based on a tight-binding chain with periodic boundary conditions, as depicted in Supplementary Figure 10(a). The Hamiltonian is:

$$H = H_{\mathrm{ring}} + H_{\mathrm{SO}} + H_{\mathrm{Z}}, \tag{7}$$

where H_{ring} contains the kinetic and chemical potential terms and H_{SO} and H_{Z} are the spin-orbit (SO) interaction and Zeeman energy terms respectively. The explicit forms of the Hamiltonians are:

$$H_{\mathrm{ring}} = (2t-\mu)\sum_{\sigma,j=1}^{N} c^{\dagger}_{j,\sigma}c_{j,\sigma} - t\sum_{\sigma,j=1}^{N}\left\{\tilde{c}^{\dagger}_{j,\sigma}\tilde{c}_{j+1,\sigma} + \mathrm{H.C.}\right\},$$

$$H_{\mathrm{SO}} = t_{\mathrm{so}}\sum_{\sigma,\sigma',j=1}^{N}\left\{-\mathrm{i}(\sigma_{\mathrm{z}})_{\sigma\sigma'}\tilde{c}^{\dagger}_{j,\sigma}\tilde{c}_{j+1,\sigma'} + \mathrm{H.C.}\right\}, \tag{8}$$

$$H_{\mathrm{Z}} = E_{\mathrm{Z}}\sum_{\sigma,\sigma',j=1}^{N}(\sigma_{\mathrm{z}})_{\sigma\sigma'}c^{\dagger}_{j,\sigma}c_{j,\sigma'},$$

where $c^{\dagger}_{j,\sigma}$ ($c_{j,\sigma}$) creates (annihilates) an electron with spin $\sigma=\uparrow,\downarrow$ on site j, μ is the chemical potential, N is the total number of sites, $t=\hbar^2/2m^*d^2$ is the kinetic energy associated with hopping between neighboring sites (m^* being the effective electron mass and d the lattice constant), $t_{\mathrm{so}}=\alpha/2d$ is the energy associated with the SO interaction (α being the SO strength) and $E_{\mathrm{Z}}=g^*_{\mathrm{spin}}\mu_{\mathrm{B}}|\mathrm{B}|/2$ is the Zeeman

energy (g^*_{spin} being the effective g-factor without orbital contributions, μ_{B} the Bohr magneton and $\mathbf{B}$ the applied magnetic field). The operators $\tilde{c}$ include the orbital magnetic field effects:

$$\tilde{c}_j = c_j \mathrm{e}^{-\mathrm{i}\frac{e\Phi}{\hbar}\frac{j}{N}}, \tag{9}$$

where e is the elementary charge, Φ is the total flux through the ring and c_j are the operators without flux contributions. The ring is divided into two QDs, left (L) and right (R), by potential barriers. We chose model parameters to obtain a good match to the experimental data and the 3D simulation. We have used $m^*_{\text{InAs,ZB}} = 0.026\, m_{\text{e}}$, $\alpha = 2.024$ meV · nm and $g^{\text{InAs}}_{\text{bulk}} = -11$. The results do not depend on the value of the chemical potential. The potential barriers separating the two QDs are 131 meV high and 3.3 nm wide. The diameter of the ring is 35 nm; this is smaller than the actual nanowire's diameter ($\simeq$ 80 nm) and the choice was made based on the observation that the ring-like states formed, have a diameter of around 1/3 – 2/3 of the nanowire's diameter (Fig. 4h in the main article).

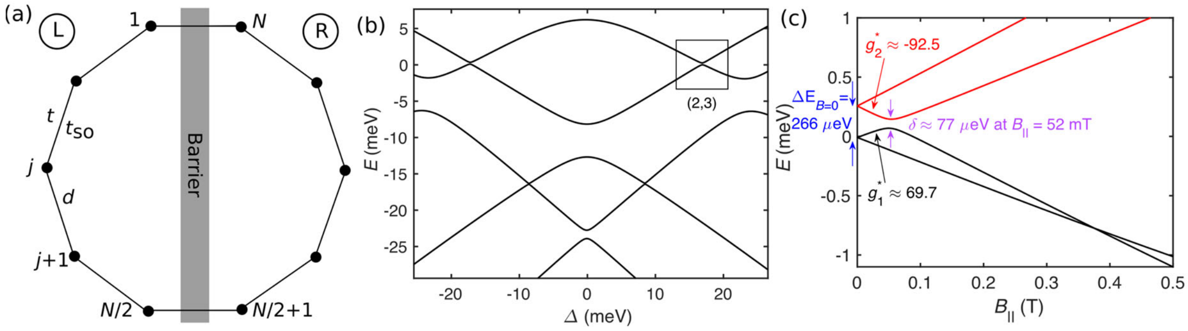

Supplementary Figure 10 **Tight-binding simulation of single-electron states, cf. Supplementary Figure 9.** (a) Pictorial representation of the model where the hoppings (t, t_{so}) and the barrier are depicted. The barrier is implemented considering higher on-site energies for sites between the sites 1, *N* and *N*/2, *N*/2+1 (not shown in the figure) and divides the ring in two half-rings, left (L) and right (R). (b) The lowest energy states as a function of the asymmetry Δ between the L and R QD at $\mathbf{B} = 0$ (the states are two-fold degenerate). The (2,3) anti-crossing, which we analyze further in (c), is shown in the black rectangle. (c) Parallel magnetic field dependence of the states involved in the (2,3) anti-crossing. The energy gap at $B_{\|} = 0$, the split at $B_{\|} = 52$ mT and the extracted g^* factors for the two Kramers pairs are denoted by blue, magenta, black and red arrows respectively and they are in good agreement with the experimental results and the 3-D nanowire simulations for the chosen parameters.

The system now consists of two half-ring QDs and applying an electric field $\mathbf{E}$ we can control which QD levels align. In practice, we include the electric field's effects by introducing an asymmetry between the on-site energies of the left and the right QDs. In Supplementary Figure 10(b) we plot the first five energy levels of the two QD system as a function of this asymmetry which we call detuning (Δ). Here $\mathbf{B} = 0$, and each energy level is 2-fold degenerate. The anti-crossings at $\Delta = 0$ correspond to situations where the same levels from each QD are aligned. For $\Delta \neq 0$, different levels align. We observe that the

energy splitting is in general larger for the (odd,odd) and (even,even) crossings than for the (odd,even) and (even,odd) ones. This is crucial to explain the formation of ring-like states and we address this point later in this section. We focus on the (2,3) anti-crossing where the large g^* values were observed experimentally, and redefine the "zero" of our detuning at $\Delta \simeq 16.916$ meV. We note that a disorder parameter of $D = 1.365$ meV was also included by adding a random energy between -1.365 and +1.365 meV at each site.

In Supplementary Figure 10(c) we plot the evolution of the energies of the states involved in the (2,3) anti-crossing with a magnetic field parallel to the nanowire axis (and thus perpendicular to the considered double QDs system, Supplementary Figure 10(a)). The energy splitting at $B_{||} = 0$ is $\Delta E_{B=0} \simeq 266\ \mu eV$ while the g^*extracted for the first and second Kramers pairs are 69.7 and -92.5 respectively. At $B_{||} = 52$ mT we observe an avoided crossing and the states split by an energy $\delta =$ 77 µeV. δ is induced by the disorder D which quantifies the quality of the ring. Thus, with the chosen parameters, the simple tight-binding chain qualitatively and quantitatively reproduces the experimental data and the 3D nanowire simulation.

The enhancement of g^* for the (2,3) anti-crossing is attributed to orbital contributions stemming from the formation of ring-like states. Calculating the expectation value of the angular momentum operator $L = \langle\Psi|\hat{\mathbf{L}}|\Psi\rangle$ for the states involved in the (2,3) anti-crossing we find $L \approx 1.075\ \hbar$, a result compatible with the "ring" picture. In contrast, $L \approx 0.01\ \hbar$ for the states involved in the (2,2) anti-crossing, where the orbital contributions are negligible. We note that a perfect ring with 7 electrons, corresponding to the 1e regime of the (2,3) crossing in terms of electron number, would have $L = 2\ \hbar$ for the electron in the outermost populated orbital.

We stress here that the extracted g^*values are very sensitive to detuning and they decrease fast away from the detuning corresponding to the (2,3) anti-crossing. This enables the manipulation of the magnetic response of the system by electrical means in a much more efficient way compared to a quantum ring without barriers. To illustrate this point, we plot the detuning dependence of g^* for a ring without barriers and for the half-ring QDs (Supplementary Figure 11). The plotted g^* is normalized by the maximum g^* value ($g^*_{\max}$) obtained for the proper detuning value for each system. We notice that for $\Delta = 1$ meV the g^* values for the QDs system have dropped to $\approx 0.1\ g^*_{\max}$ and $\approx 0.3\ g^*_{\max}$ for the lower and upper Kramers pair respectively, whereas g^*_{ring} is practically unaffected and we would have to go to much larger Δ values to observe a decrease. We conclude that even though large effective g-factors can also be extracted for a ring without barriers, the two half-ring QD system is significantly more favorable for electrical manipulation of g^*.

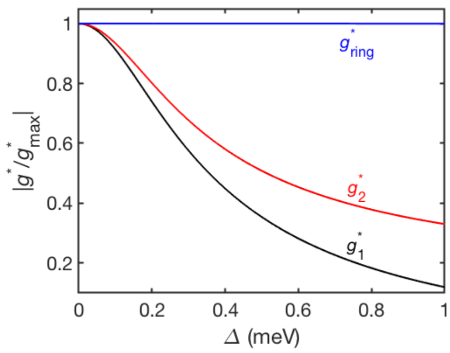

Supplementary Figure 11 Detuning dependence of the normalized g^* for a ring without barriers (blue) and for the system of two half-ring QDs (black for lower and red for upper Kramers pair). g^*_{ring} is practically unaffected for the depicted range of detuning, while g^*_1 and g^*_2 decay fast.

We now turn to a perturbation theory analysis to gain further insight into why the ring-like states only form for the (even,odd) and (odd,even) crossings. The unperturbed system consists of completely separated L and R QDs, which are one-dimensional quantum wells, their states being described by the usual sinusoidal wave functions. In the TB description the wave functions for the L QD can be written as:

$$|\Psi^{(0)}_{\mathrm{L},n}\rangle = \sum_{\sigma,j=1}^{N/2} a_{\mathrm{L},n,j,\sigma}|\phi_{\mathrm{L},j,\sigma}\rangle, \tag{10}$$

where $|\phi_{\mathrm{L},j,\sigma}\rangle$, $a_{\mathrm{L},n,j,\sigma}$ are the wavefunction and the wavefunction coefficient at site j and for spin component $\sigma =\uparrow,\downarrow$ and n is the number of the state in the QD. Similarly, for the R QD:

$$|\Psi^{(0)}_{\mathrm{R},n}\rangle = \sum_{\sigma,j=\frac{N}{2}+1}^{N} a_{\mathrm{R},n,j,\sigma}|\phi_{\mathrm{R},j,\sigma}\rangle. \tag{11}$$

We note that the standard particle in a box wave functions obey:

$$\begin{aligned}
a_{\mathrm{L},n,1,\sigma} &= a_{\mathrm{L},n,N/2,\sigma} = a \qquad &&\text{for } n \text{ odd},\\
a_{\mathrm{L},n,1,\sigma} &= -a_{\mathrm{L},n,N/2,\sigma} = a \qquad &&\text{for } n \text{ even},\\
a_{\mathrm{R},n,\frac{N}{2}+1,\sigma} &= a_{\mathrm{R},n,N,\sigma} = a \qquad &&\text{for } n \text{ odd},\\
a_{\mathrm{R},n,\frac{N}{2}+1,\sigma} &= a_{\mathrm{R},n,N,\sigma} = a \qquad &&\text{for } n \text{ even},
\end{aligned} \tag{12}$$

where a can be chosen real and positive.

When two levels in the double QDs system are aligned, they form a degenerate subspace. We now consider a perturbation in the form of a coupling between the sites 1, N and $N/2$, $N/2+1$

$$\delta\mathrm{H} = -t\left\{c^{\dagger}_{\frac{N}{2},\uparrow}c_{\frac{N}{2}+1,\uparrow} + c^{\dagger}_{\frac{N}{2},\downarrow}c_{\frac{N}{2}+1,\downarrow} + c^{\dagger}_{N,\uparrow}c_{1,\uparrow} + c^{\dagger}_{N,\downarrow}c_{1,\downarrow} + \mathrm{H.C.}\right\}$$

$$+t_{\mathrm{SO}}\left\{-ic^{\dagger}_{\frac{N}{2},\uparrow}c_{\frac{N}{2}+1,\uparrow} + ic^{\dagger}_{\frac{N}{2},\downarrow}c_{\frac{N}{2}+1,\downarrow} - ic^{\dagger}_{N,\uparrow}c_{1,\uparrow} + ic^{\dagger}_{N,\downarrow}c_{1,\downarrow} + \mathrm{H.C.}\right\}, \quad (13)$$

according to (S8). At **B** = 0 the spin states are degenerate and in the following we focus on one Kramers pair, choosing to work with spin up, as the spin degeneracy cannot be broken with the considered perturbation.

For the (2,2) anti-crossing the aligned states are $|\Psi^{(0)}_{\mathrm{L},2}\rangle$ and $|\Psi^{(0)}_{\mathrm{R},2}\rangle$, thus $E^{0}_{\mathrm{L},2} = E^{0}_{\mathrm{R},2} = E^{(0)}_{(2,2)}$. For the matrix elements of δH in the degenerate subspace we find:

$$\delta\mathrm{H}_{11} = \left\langle\Psi^{(0)}_{\mathrm{L},2}\right|\delta\mathrm{H}\left|\Psi^{(0)}_{\mathrm{L},2}\right\rangle = 0$$

$$\delta\mathrm{H}_{12} = \left\langle\Psi^{(0)}_{\mathrm{L},2}\right|\delta\mathrm{H}\left|\Psi^{(0)}_{\mathrm{R},2}\right\rangle = -a^2(-t) - a^2(-t) = 2a^2t$$

$$\delta\mathrm{H}_{21} = \left\langle\Psi^{(0)}_{\mathrm{R},2}\right|\delta\mathrm{H}\left|\Psi^{(0)}_{\mathrm{L},2}\right\rangle = -a^2(-t) - a^2(-t) = 2a^2t$$

$$\delta\mathrm{H}_{22} = \left\langle\Psi^{(0)}_{\mathrm{R},2}\right|\delta\mathrm{H}\left|\Psi^{(0)}_{\mathrm{R},2}\right\rangle = 0. \quad (14)$$

Diagonalizing δH we obtain the first order energy corrections and the corresponding linear combinations of the unperturbed wavefunctions:

$$\left|\Psi^{(0)}_{\alpha,(2,2)}\right\rangle = \frac{1}{\sqrt{2}}\left(\left|\Psi^{(0)}_{\mathrm{L},2}\right\rangle - |\Psi^{(0)}_{\mathrm{R},2}\rangle\right), \qquad E^{(1)}_{\alpha,(2,2)} = E^{(0)}_{(2,2)} - 2a^2t,$$

$$\left|\Psi^{(0)}_{\beta,(2,2)}\right\rangle = \frac{1}{\sqrt{2}}\left(\left|\Psi^{(0)}_{\mathrm{L},2}\right\rangle + |\Psi^{(0)}_{\mathrm{R},2}\rangle\right), \qquad E^{(1)}_{\beta,(2,2)} = E^{(0)}_{(2,2)} + 2a^2t. \quad (15)$$

We follow a similar procedure for the (2,3) anti-crossing. Now $E^{0}_{\mathrm{L},2} = E^{0}_{\mathrm{R},3} = E^{(0)}_{(2,3)}$, since the aligned states are $|\Psi^{(0)}_{\mathrm{L},2}\rangle$ and $|\Psi^{(0)}_{\mathrm{R},3}\rangle$. The first order energy corrections and the corresponding linear combination of the unperturbed wavefunctions are:

$$\left|\Psi^{(0)}_{\alpha,(2,3)}\right\rangle = \frac{1}{\sqrt{2}}\left(\left|\Psi^{(0)}_{\mathrm{L},2}\right\rangle - \mathrm{i}|\Psi^{(0)}_{\mathrm{R},3}\rangle\right), \qquad E^{(1)}_{\alpha,(2,3)} = E^{(0)}_{(2,3)} + 2a^2t_{\mathrm{SO}},$$

$$\left|\Psi^{(0)}_{\beta,(2,3)}\right\rangle = \frac{1}{\sqrt{2}}\left(\left|\Psi^{(0)}_{\mathrm{L},2}\right\rangle + \mathrm{i}|\Psi^{(0)}_{\mathrm{R},3}\rangle\right), \qquad E^{(1)}_{\beta,(2,3)} = E^{(0)}_{(2,3)} - 2a^2t_{\mathrm{SO}}. \quad (16)$$

The above results can explain the large difference between the (2,2) and (2,3) energy splittings visible in Supplementary Figure 10(b). In the (2,2) case the splitting is $4a^2t$ which is much larger than $4a^2t_{\mathrm{SO}}$

in the (2,3) case. The (even,odd) splittings are thus induced by t_{SO} and vanish for $t_{\mathrm{SO}} = 0$. From the TB results we can extract $4a^2 t \doteq 4\tilde{t} = 4.528\,\mathrm{meV}$ and $4a^2 t_{\mathrm{SO}} \doteq 4\tilde{t}_{\mathrm{SO}} = 0.266\,\mathrm{meV}$. It is now straightforward to write down the first order corrections to the wave functions (up to a normalization factor):

$$\left|\Psi^{(1)}_{\alpha(\beta),(2,2)}\right\rangle = \left|\Psi^{(0)}_{\alpha(\beta),(2,2)}\right\rangle \pm \sqrt{2}\left\{\tilde{t}\sum_p \frac{1}{E^{(0)}_{p_{\mathrm{LE}}} - E^{(0)}_{(2,2)}}\left|p_{\mathrm{LE}}{}^{(0)}\right\rangle - \mathrm{i}\tilde{t}_{\mathrm{so}}\sum_p \frac{1}{E^{(0)}_{p_{\mathrm{LO}}} - E^{(0)}_{(2,2)}}\left|p_{\mathrm{LO}}{}^{(0)}\right\rangle\right\}$$

$$-\sqrt{2}\left\{\tilde{t}\sum_p \frac{1}{E^{(0)}_{p_{\mathrm{RE}}} - E^{(0)}_{(2,2)}}\left|p_{\mathrm{RE}}{}^{(0)}\right\rangle - \mathrm{i}\tilde{t}_{\mathrm{so}}\sum_p \frac{1}{E^{(0)}_{p_{\mathrm{RO}}} - E^{(0)}_{(2,2)}}\left|p_{\mathrm{RO}}{}^{(0)}\right\rangle\right\}, \qquad (17)$$

$$\left|\Psi^{(1)}_{\alpha(\beta),(2,3)}\right\rangle = \left|\Psi^{(0)}_{\alpha(\beta),(2,3)}\right\rangle \mp \sqrt{2}\left\{\tilde{t}_{\mathrm{so}}\sum_p \frac{1}{E^{(0)}_{p_{\mathrm{LE}}} - E^{(0)}_{(2,3)}}\left|p_{\mathrm{LE}}{}^{(0)}\right\rangle - \mathrm{i}\tilde{t}\sum_p \frac{1}{E^{(0)}_{p_{\mathrm{LO}}} - E^{(0)}_{(2,3)}}\left|p_{\mathrm{LO}}{}^{(0)}\right\rangle\right\}$$

$$-\sqrt{2}\left\{\tilde{t}\sum_p \frac{1}{E^{(0)}_{p_{\mathrm{RE}}} - E^{(0)}_{(2,3)}}\left|p_{\mathrm{RE}}{}^{(0)}\right\rangle - \mathrm{i}\tilde{t}_{\mathrm{so}}\sum_p \frac{1}{E^{(0)}_{p_{\mathrm{RO}}} - E^{(0)}_{(2,3)}}\left|p_{\mathrm{RO}}{}^{(0)}\right\rangle\right\}$$

$$\mp\frac{1}{2}\left\{\frac{\tilde{t}^2}{\tilde{t}_{\mathrm{so}}}\sum_p \frac{1}{E^{(0)}_{p_{\mathrm{RE}}} - E^{(0)}_{(2,3)}} + \tilde{\mathrm{t}}_{so}\sum_p \frac{1}{E^{(0)}_{p_{\mathrm{RO}}} - E^{(0)}_{(2,3)}}\right\}\left|\Psi^{(0)}_{\beta(\alpha),(2,3)}\right\rangle$$

$$\mp\frac{1}{2}\left\{-\tilde{t}_{\mathrm{so}}\sum_p \frac{1}{E^{(0)}_{p_{\mathrm{LE}}} - E^{(0)}_{(2,3)}} - \frac{\tilde{t}^2}{\tilde{t}_{\mathrm{so}}}\sum_p \frac{1}{E^{(0)}_{p_{\mathrm{LO}}} - E^{(0)}_{(2,3)}}\right\}\left|\Psi^{(0)}_{\beta(\alpha),(2,3)}\right\rangle, \qquad (18)$$

where index $p_{\mathrm{L(R)E(O)}}$ refers the p^{th}even (odd) state outside the degenerate subspace in QD L (R). Examining the above expressions we notice that in the (2,2) case even and odd wave functions are added multiplied with $\tilde{t}$ and $\mathrm{i}\tilde{t}_{\mathrm{so}}$ respectively. Adding odd purely imaginary wavefunctions to the even $\left|\Psi^{(0)}_{\alpha(\beta),(2,2)}\right\rangle$ contributes in making the zeros in the magnitude of $\left|\Psi^{(1)}_{\alpha(\beta),(2,2)}\right\rangle$ finite, but since $\tilde{t}_{\mathrm{so}} \ll \tilde{t}$ the effect is not noticeable. The situation is different in the (2,3) case. Odd imaginary wave functions are added to $\left|\Psi^{(0)}_{\mathrm{L},2}\right\rangle$ and even real wavefunctions are added to $\mathrm{i}|\Psi^{(0)}_{\mathrm{R},3}\rangle$, in both cases multiplied with $\tilde{t}$. This has the effect that the magnitude of $\left|\Psi^{(1)}_{\alpha(\beta),(2,3)}\right\rangle$ varies less in real space. In this sense the wave functions in the (2,3) case are more like the eigenstates of a perfect ring, which have a constant magnitude.

Supplementary References

1. Winkler, R. *Spin-Orbit Coupling Effects in Two-Dimensional Electron and Hole Systems. Transport Book* **191,** (Springer Berlin Heidelberg, 2003).

2. Manchon, A., Koo, H. C., Nitta, J., Frolov, S. M. & Duine, R. A. New perspectives for Rashba spin–orbit coupling. *Nat. Mater.* **14,** 871–882 (2015).

3. Chen, I.-J. *et al.* Conduction Band Offset and Polarization Effects in InAs Nanowire Polytype Junctions. *Nano Lett.* **17,** 902–908 (2017).

4. Winkler, G. W. *et al.* Orbital Contributions to the Electron g- Factor in Semiconductor Nanowires. *Phys. Rev. Lett.* **119,** 037701 (2017).

5. Belabbes, A., Panse, C., Furthmüller, J. & Bechstedt, F. Electronic bands of III-V semiconductor polytypes and their alignment. *Phys. Rev. B* **86,** 1–12 (2012).

6. Faria Junior, P. E. *et al.* Realistic multiband k·p approach from ab initio and spin-orbit coupling effects of InAs and InP in wurtzite phase. *Phys. Rev. B* **93,** 235204 (2016).
